# Mouse Liver-Expressed Shiftless Is an Evolutionarily Conserved Antiviral Effector Restricting Human and Murine Hepaciviruses

**DOI:** 10.1128/spectrum.01284-23

**Published:** 2023-06-21

**Authors:** Yudi Zhang, Volker Kinast, Julie Sheldon, Nicola Frericks, Daniel Todt, Matthias Zimmer, Neva Caliskan, Richard J. P. Brown, Eike Steinmann, Thomas Pietschmann

**Affiliations:** a Institute for Experimental Virology, TWINCORE Centre for Experimental and Clinical Infection Research, Hannover, Germany; b Department for Molecular and Medical Virology, Ruhr University Bochum, Bochum, Germany; c Department of Medical Microbiology and Virology, Carl von Ossietzky University Oldenburg, Oldenburg, Germany; d European Virus Bioinformatics Center (EVBC), Jena, Germany; e Helmholtz Institute for RNA-based Infection Research (HIRI), Helmholtz Zentrum für Infektionsforschung (Helmholtz Centre for Infection Research), Würzburg, Germany; f University of Würzburg, Faculty of Medicine, Würzburg, Germany; g Division of Veterinary Medicine, Paul Ehrlich Institute, Langen, Germany; h Cluster of Excellence RESIST (EXC 2155), Hannover Medical School, Hannover, Germany; i German Center for Infection Research (DZIF), Partner Site Hannover-Braunschweig, Hannover, Germany; Cornell University College of Veterinary Medicine

**Keywords:** restriction factor, HCV, liver, IFN, species tropism, virus, hepatitis C virus

## Abstract

Mice are refractory to infection with human-tropic hepatitis C virus (HCV), although distantly related rodent hepaciviruses (RHV) circulate in wild rodents. To investigate whether liver intrinsic host factors can exhibit broad restriction against these distantly related hepaciviruses, we focused on Shiftless (*Shfl*), an interferon (IFN)-regulated gene (IRG) which restricts HCV in humans. Unusually, and in contrast to selected classical IRGs, human and mouse SHFL orthologues (hSHFL and mSHFL, respectively) were highly expressed in hepatocytes in the absence of viral infection, weakly induced by IFN, and highly conserved at the amino acid level (>95%). Replication of both HCV and RHV subgenomic replicons was suppressed by ectopic expression of mSHFL in human or rodent hepatoma cell lines. Gene editing of endogenous *mShfl* in mouse liver tumor cells increased HCV replication and virion production. Colocalization of mSHFL protein with viral double-stranded RNA (dsRNA) intermediates was confirmed and could be ablated by mutational disruption of the SHFL zinc finger domain, concomitant with a loss of antiviral activity. In summary, these data point to an evolutionarily conserved function for this gene in humans and rodents: SHFL is an ancient antiviral effector which targets distantly related hepaciviruses via restriction of viral RNA replication.

**IMPORTANCE** Viruses have evolved ways to evade or blunt innate cellular antiviral mechanisms within their cognate host species. However, these adaptations may fail when viruses infect new species and can therefore limit cross-species transmission. This may also prevent development of animal models for human-pathogenic viruses. HCV shows a narrow species tropism likely due to distinct human host factor usage and innate antiviral defenses limiting infection of nonhuman liver cells. Interferon (IFN)-regulated genes (IRGs) partially inhibit HCV infection of human cells by diverse mechanisms. Here, we show that mouse Shiftless (mSHFL), a protein that interferes with HCV replication factories, inhibits HCV replication and infection in human and mouse liver cells. We further report that the zinc finger domain of SHFL is important for viral restriction. These findings implicate mSHFL as a host factor that impairs HCV infection of mice and provide guidance for development of HCV animal models needed for vaccine development.

## INTRODUCTION

Discovered in 1989 (1), hepatitis C virus (HCV) represents a major human pathogen, causing liver fibrosis, cirrhosis, and hepatocellular carcinoma (HCC) (2). The World Health Organization (WHO) estimates that more than 58 million people are chronically infected with HCV, with approximately 1.5 million new infections occurring annually (3). The introduction of direct-acting antiviral (DAA) therapies successfully eliminates HCV infection in >90% of patients (4). However, limited screening capacity and access to DAA treatment in the developing world, together with the lack of an available vaccine, impede the global eradication of HCV (5).

HCV has a narrow host range: only humans and chimpanzees can be robustly infected with the virus. Since chimpanzees are no longer used as an animal model for HCV (1, 6), and despite the fact that several efforts have been made toward developing suitable models for HCV research, so far no tractable *in vivo* model for HCV research is available (7).

Over the last decade, a range of hepaciviruses have been discovered, including equine hepacivirus (EqHV) and bovine hepacivirus (BovHV), both causing acute or chronic infections in horses and cattle, respectively (8, 9). Other hepaciviruses in smaller mammals have also been recently discovered, including the Norway rat hepacivirus (NrHV), which can infect laboratory rats (10). These viruses offer new opportunities to study the pathophysiology of hepacivirus infections *in vivo*. Although they can serve as important surrogate models, they do not satisfy the need for an *in vivo* model for HCV infection.

To render mice susceptible to HCV infection, both ectopic expression of human entry factors occludin (OCLN) and CD81 and ablation of interferon (IFN)-based antiviral innate immune responses are required (11). Moreover, Scull et al. reported transient HCV viremia in rhesus macaque liver cells, provided their innate immune response was blunted by inhibition of IFN signaling (12). Thus, the narrow host tropism of HCV is at least in part caused by IFN-dependent expression of antiviral restriction factors.

HCV infection induces vigorous innate immune responses characterized by upregulation of numerous IFN-regulated genes (IRGs) (13–16). In addition, several IRGs have been shown to inhibit HCV infection of human cells (17–19). One of these is Shiftless (*SHFL*; also called *SFL*, *C19orf66*, *RyDEN*, or *IRAV*), an IRG that inhibits multiple viruses, including dengue virus (DENV), HCV, human immunodeficiency virus (HIV), Kaposi’s sarcoma-associated herpesvirus (KSHV), herpes simplex virus 1 (HSV-1), chikungunya virus (CHIKV), Zika virus (ZIKV), and West Nile virus (WNV) (20–26). Recently, we reported that *Shfl* is upregulated in liver biopsy specimens of chronically HCV-infected patients and noted that ectopic expression of SHFL in a human hepatoma cell line suppressed HCV replication by disturbing the formation of viral replication factories (19). In the present study, we examined if SHFL orthologues from diverse species inhibit HCV and specifically focused on Mus musculus SHFL (mSHFL). We noted that ectopic expression of mSHFL reduced frameshifting levels during translation of the viral RNA and observed that mSHFL inhibited HCV and rodent hepacivirus replication in murine cells.

## RESULTS

### SHFL is a highly expressed IRG and highly conserved between human and mouse.

**FIG 1:**
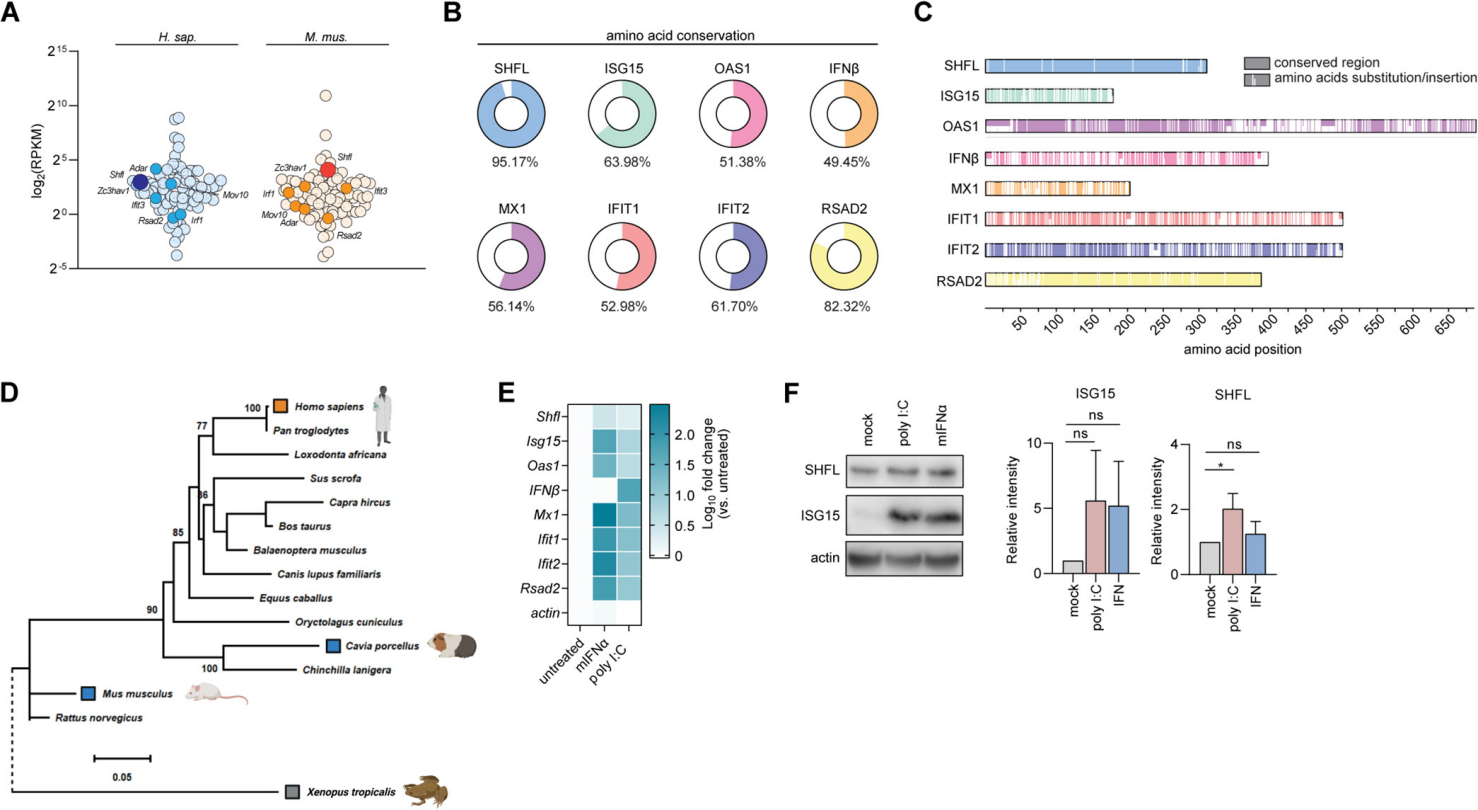
*Shfl* is a highly expressed and highly conserved interferon-regulated gene (IRG). (A) Intrinsic mRNA expression levels of selected IRGs (*n* = 89) in primary human hepatocytes (*n* = 3) and total mouse livers (*n* = 3). Presented IRGs are defined as core IRGs by Shaw et al. (27). Transcriptome sequencing (RNA-seq) data were extracted from transcriptomic profiling results recently published by Brown et al. (32). (B and C) Amino acid conservation between human and mouse IRG orthologues. Colored bars indicate the conserved residues, while white bars represent residues that differ in mouse orthologues relative to human amino acid sequences. Percentages in panel B represent the amino acid conservation of mouse proteins with their human counterparts. (D) Species-specific differences in SHFL evolution. A phylogenetic tree depicts the evolutionary relationship of SHFL orthologues. Significant bootstrap values (>70%) are indicated. Species orthologues used in this investigation were highlighted on the tree using Biorender. (E) Modulation of *Shfl* mRNA expression in PMH induced by different stimuli. The gradient color of blue indicates log_10_ fold change of *M. musculus Shfl* (*mShfl*) and other characterized IRGs in plated PMH 8 h after treatment with mouse IFN-α (1,000 U/mL) or poly(I·C) (50 μg/mL). (F) Immunoblot analysis of SHFL protein expression in PMH induced by different stimuli. Plated PMH were treated with mouse IFN-α (1,000 U/mL) or poly(I:C) (50 μg/mL) for 8 h and subjected to immunoblot analysis for SHFL and ISG15 protein expression. Depicted is a representative immunoblot (*n* = 3). Data from 3 independent experiments are shown as means + SDs (one-way ANOVA, Dunnett’s multiple-comparison test; *, *P* < 0.05; ns, nonsignificant).

Multiple studies have addressed the antiviral strategies of host cells to combat viral infections. Considering that innate immunity likely contributes to HCV’s narrow host tropism, we reasoned that intrinsically expressed restriction factors may be relevant in shaping species barriers. Therefore, we compared the baseline expression of a panel of 89 core IRGs (27) in primary human hepatocytes (PHH) and in total livers from different standard laboratory mouse strains (*n* = 3). We observed that SHFL exhibited high baseline expression in the liver compared to other IRGs with reported anti-HCV activity (Fig. 1A). To investigate rates of evolutionary conservation between human and mouse antiviral effectors, we aligned protein sequences of selected human and mouse IRG orthologues (Fig. 1B). Surprisingly, the human-mouse SHFL alignment demonstrated an unexpectedly high level (95.17%) of amino acid conservation (in terms of conservative replacements). In contrast, comparison of human and mouse orthologues from our control panel of seven IRGs known to be upregulated during virus infection (28) revealed lower levels of amino acid conservation between human and mouse proteins (Fig. 1B and C). Together, these data demonstrate high baseline expression of SHFL in the murine liver and unexpectedly high levels of conservation between human and mouse protein orthologues.

To examine the evolutionary relationships of *Shfl* sequences from divergent species, phylogenetic analysis was performed on *Shfl* gene orthologues from 15 different species, including another rodent and an evolutionarily more distant species: guinea pig (Cavia porcellus) and tropical clawed frog (Xenopus tropicalis) (Fig. 1D). The C. porcellus *Shfl* gene was distinct from *hShfl*. Interestingly, the *mShfl* gene did not cluster closely with C. porcellus *Shfl* but rather branched off earlier from the lineage containing humans and nonmurine rodents. While hSHFL and mSHFL are highly conserved at the amino acid level, closer inspection of the underlying nucleotide sequences revealed high numbers of synonymous changes between the two species, contributing to the large genetic distance observed between human and mouse in the phylogenetic tree (Fig. 1C and D). These observations indicate that hSHFL and mSHFL are subject to strong purifying selection, likely due to shared functional constraints on protein evolution.

Next, to characterize the regulation of *Shfl* by IFN, we compared the mRNA levels of *Shfl* in primary mouse hepatocytes (PMH) from C57BL/6 mice. PMH were treated with either mouse IFN-α or double-stranded RNA (dsRNA) mimic poly(I:C), and the change in gene expression was compared to mock-treated controls. *Shfl* was significantly upregulated in response to mouse IFN-α and poly(I:C) treatment. Control IRGs, *Isg15*, *Oas1*, *Mx1*, *Ifit1*, *Ifit2*, and *Rsad2*, were strongly induced upon both stimuli, while the mRNA of *Ifnb1* was upregulated only when PMH were treated with poly(I:C) (Fig. 1E). To investigate the modulation of SHFL expression at the protein level, we subjected mouse IFN-α-, poly(I:C)-, or mock-treated PMH to Western blot analysis. While not statistically significant, robust induction of ISG15 protein levels was detected upon both stimuli compared with that in mock-treated PMH. In contrast, SHFL was significantly upregulated in the presence of poly(I:C) (Fig. 1F). Taken together, these data confirm baseline SHFL protein expression in murine hepatocytes and demonstrate that specific immune stimuli can modestly enhance expression of SHFL.

### mSHFL restricts HCV infection in human hepatoma cells.

**FIG 2:**
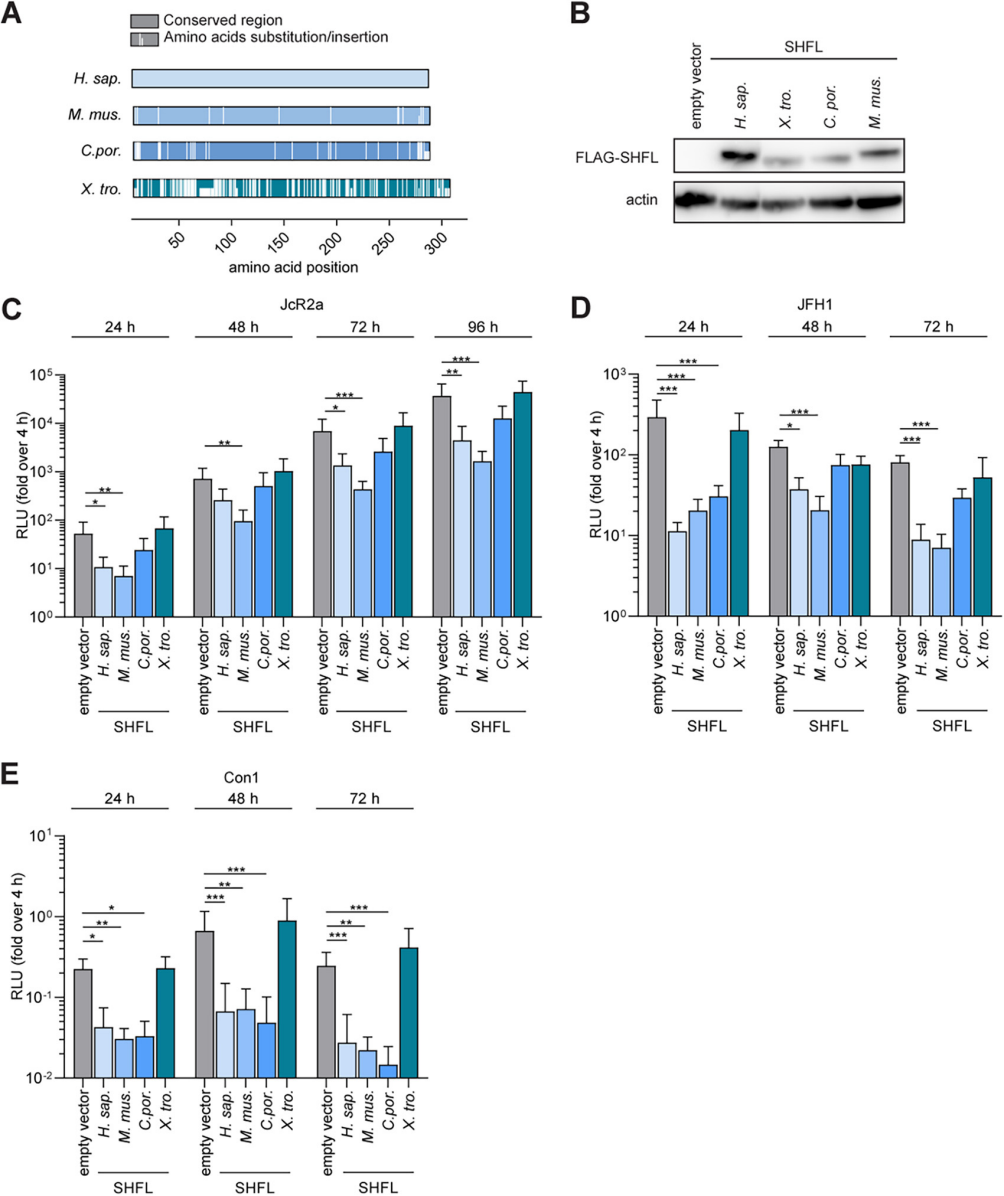
mSHFL restricts HCV infection in human hepatoma cells. (A) Amino acid conservation between SHFL orthologues from different species. Colored bars indicate the conserved residues, while white bars represent residues that differ relative to human amino acid sequences. (B) Western blot of Huh-7.5 cell lines stably expressing empty vector or SHFL orthologues. (C) HCV JcR2a infection in Huh-7.5 cell lines expressing SHFL orthologues. *Renilla* luciferase reporter expression was measured (relative light units [RLU]) and normalized to empty vector at 4 h postinfection. Data from 5 independent experiments are shown as means + SDs (two-way ANOVA, Dunnett’s multiple-comparison test; *, *P* < 0.05; **, *P* < 0.01; ***, *P* < 0.001). (D) HCV JFH1 SGR replication in Huh-7.5 cell lines expressing SHFL orthologues. (E) HCV Con1 SGR replication in Huh-7.5 cell lines expressing SHFL orthologues. For panels D and E, firefly luciferase reporter activity was measured and normalized to empty vector at 4 h posttransfection. Data from 3 independent experiments are shown as means + SDs (two-way ANOVA, Dunnett’s multiple-comparison test; *, *P* < 0.05; **, *P* < 0.01; ***, *P* < 0.001). *H. sap.*, Homo sapiens; *M. mus*., Mus musculus; *C. por.*, Cavia porcellus; *X. tro.*, *Xenopus tropicalis*.

To evaluate the antiviral function of SHFL orthologues, we selected SHFL from 4 species (highlighted in Fig. 1D), including humans (Homo sapiens), tropical clawed frogs (X. tropicalis), guinea pigs (C. porcellus), and mice (M. musculus), with a wide range of amino acid sequence homologies compared to hSHFL (from 95.17% to 52.63%) (Fig. 2A). We then generated Huh-7.5 cell lines expressing either the selected SHFL orthologues tagged with a triple-FLAG tag or an empty-vector control. We validated the expression of these proteins by Western blotting (Fig. 2B) and evaluated their influence on HCV infection using the HCV JcR2a *Renilla* luciferase reporter virus (29). We observed that ectopically expressed hSHFL restricted HCV replication 10-fold at 96 h postinfection (h p.i.). In a similar manner, mSHFL restricted replication 22-fold, whereas C. porcellus SHFL expression resulted in a 3-fold but nonsignificant reduction of HCV replication. The most distantly related SHFL, X. tropicalis SHFL, displayed no HCV restriction (Fig. 2C). We previously reported that hSHFL restricted HCV RNA replication by interference with the production of viral RNA replication factories (19). To investigate whether mSHFL inhibits HCV RNA replication, we transfected two different HCV subgenomic replicon (SGR) transcripts from GT2a (JFH1) and GT1b (Con 1) isolates into Huh-7.5 cell lines expressing SHFL orthologues and investigated the SGR replication fitness (Fig. 2D and E). The results revealed that in the presence of hSHFL, mSHFL, or C. porcellus SHFL in Huh-7.5 cells, replication of both GT2a and GT1b SGRs was suppressed. In summary, our data suggest that mSHFL is capable of inhibiting human-tropic HCV replication.

### mSHFL restricts HCV infection in mouse liver tumor cells.

**FIG 3:**
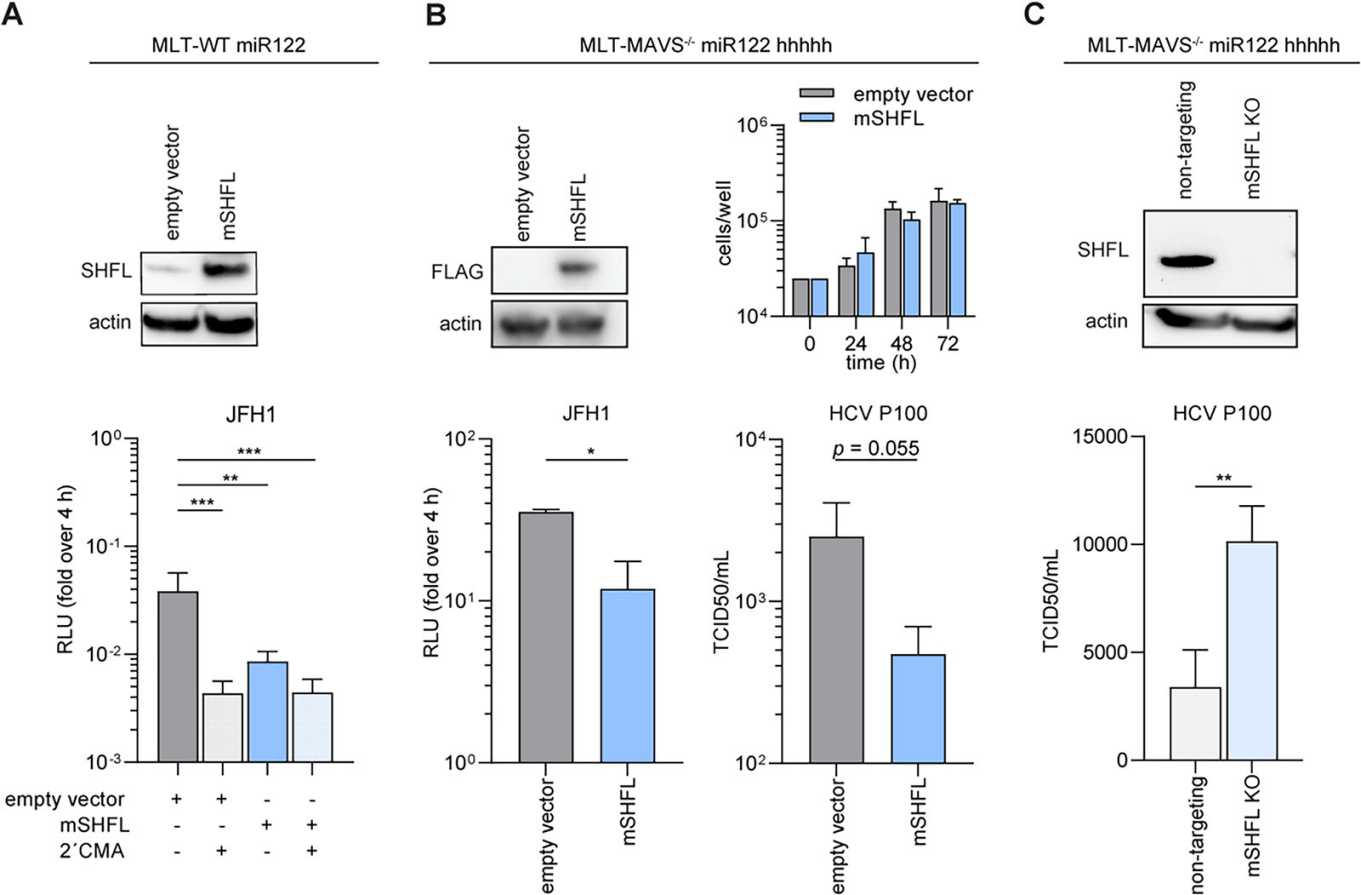
*M. musculus* SHFL (mSHFL) restricts HCV infection in murine cells. (A) (Top) Western blot of MLT-WT miR-122 cell lines stably expressing empty vector or mSHFL. (Bottom) HCV JFH1 SGR replication in MLT wt-miR122 cells expressing mSHFL. Firefly luciferase reporter activity was measured and normalized to empty vector counts at 4 h posttransfection. The nucleotide analogue 2′C-methyl-adenosine (2′CMA) served as a control for inhibition of HCV replication. Data from 3 independent experiments are shown as means + SDs (two-way ANOVA, Dunnett’s multiple-comparison test; *, *P* < 0.05; **, *P* < 0.01; ***, *P* < 0.001). (B) (Top left) Western blot of MLT- MAVS^−/−^ miR-122 hhhhh cell lines stably expressing empty vector or mSHFL. (Top right) Potential antiproliferative properties of SHFL were assessed via visual cell counting after trypan blue staining and compared to empty vector control cells. Data from 3 independent experiments are shown as means + SDs. (Bottom left) HCV JFH1 SGR replication in MLT- MAVS^−/−^ miR-122 hhhhh cells stably expressing mSHFL. Firefly luciferase reporter activity was measured 48 h posttransfection and normalized to empty vector counts at 4 h posttransfection. Data from 3 independent experiments are shown as means + SDs (unpaired two-tailed *t* test; *, *P* < 0.05). (Bottom right) HCVcc P100 infection of MLT- MAVS^−/−^ miR-122 hhhhh cell lines stably expressing an empty vector or mSHFL. Infectivity was calculated via limiting dilution assays to determine 50% tissue culture infective doses (TCID_50_) per milliliter at 48 h postinfection. Data from 3 independent experiments are shown as means + SDs (unpaired two-tailed *t* test; *, *P* < 0.05). (C) (Top) Western blot analysis of nontargeted and *mShfl* gene-edited MLT-MAVS^−/−^ miR-122 hhhhh cells. (Bottom) HCVcc P100 infection of nontargeted and *mShfl* gene-edited MLT-MAVS^−/−^ miR-122 hhhhh cells. Infectivity was calculated via limiting-dilution assays to determine TCID_50_ per milliliter at 72 h postinfection. Data from 3 independent experiments are shown as means + SDs (unpaired two-tailed *t* test; **, *P* < 0.01).

Since ectopic expression of mSHFL in human Huh-7.5 cells restricted HCV replication, we next asked if we could observe a similar phenotype in mouse liver cells and whether this HCV restriction capacity could limit cross-species transmission of HCV to mice. Thus, first we expressed mSHFL in mouse liver tumor cells which express miR-122 (MLT-WT miR-122), since these cells have previously been shown to be permissive for HCV RNA replication (Fig. 3A) (30). HCV SGR JFH1 was transfected into mSHFL cells and replication fitness was assessed with the nucleotide analogue 2′C-methyl-adenosine (2′CMA) serving as a control. Here, we observed a reduction of JFH1 SGR replication in the presence of mSHFL compared to the empty-vector control, confirming the restriction of HCV replication by mSHFL in mouse liver tumor cells (Fig. 3A).

Given the modest replication efficiency of HCV in MLT-WT miR-122 cells, we next utilized another mouse liver tumor cell line that robustly supports HCV infection due to ectopic expression of human entry factors (MLT-MAVS^−/−^ miR-122 hhhhh) (30). Ectopic expression of mSHFL was validated via Western blot analysis (Fig. 3B). Subsequent experiments revealed that mSHFL restricted HCV SGR JFH1 replication in these cells without affecting cell viability (Fig. 3B). To assess whether mSHFL is capable of restricting the whole HCV life cycle, ranging from initial entry to final release of progeny virus, we took advantage of an HCV population with high replicative fitness, cell culture-adapted HCV (HCVcc) P100 (31). This virus has been shown to exhibit enhanced replicative capacity in MLT cells (32). Quantification of progeny virus revealed lower progeny virus titers in the presence of mSHFL (Fig. 3B), suggesting that mSHFL is capable of preventing HCV propagation in murine cells.

To determine whether endogenous SHFL contributes to the restriction of HCV in murine cells, we performed gene editing of *Shfl* by utilizing CRISPR/Cas9 technology. As determined by Western blotting, *Shfl* gene-edited cell populations exhibited reduced SHFL expression compared with the nontargeting control (Fig. 3C). We subsequently evaluated HCV replication and observed that infectious virus release was up to 2-fold higher in the *Shfl* gene-edited cell populations than in nontargeting cells (Fig. 3C). Taken together, these data suggest that ectopic and endogenous mSHFL modestly inhibits HCV SGR replication and virion production of a cell culture-adapted HCV strain in a murine cellular context.

### mSHFL colocalizes with viral dsRNA.

**FIG 4:**
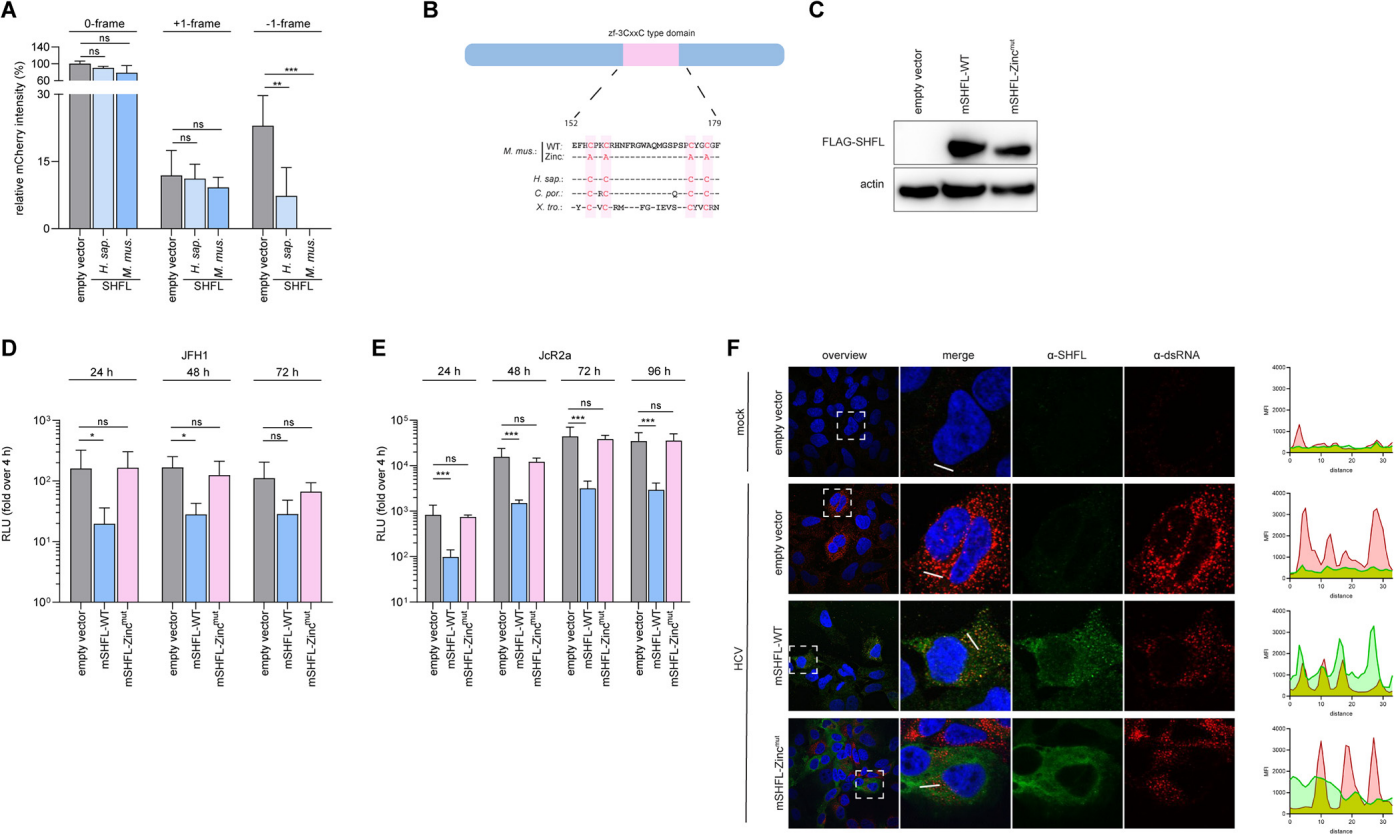
mSHFL colocalizes with viral dsRNA upon HCV infection. (A) Modulation of ribosomal frameshifting by mSHFL. Huh-7.5 cells ectopically expressing hSHFL or mSHFL were transfected with a frameshift reporter construct; the PRF efficiency was determined by expression level of EGFP and/or mCherry. Data were normalized to Huh-7.5 cells expressing empty vector as a negative control. Data from 3 independent experiments are shown as means + SDs (one-way ANOVA, Dunnett’s multiple-comparison test; *, *P* < 0.05; **, *P* < 0.01; ***, *P* < 0.001). (B) Schematic illustration of predicted zinc finger domains and their amino acid sequences of different SHFL orthologues. Black bars illustrate the same amino acid sequence, while the blank positions indicate gaps in the coding sequence; the positions of the cysteine-to-alanine substitutions in the zinc finger mutant used in this study are highlighted in red. (C) Western blot of Huh-7.5 cell lines stably expressing empty vector, mSHFL-WT, or mSHFL-Zinc^mut^. (D) Replication of the HCV JFH1 SGR in Huh-7.5 cell lines expressing mSHFL-WT or mSHFL-Zinc^mut^. (E) HCV JcR2a infection in Huh-7.5 cell lines expressing mSHFL-WT or mSHFL-Zinc^mut^. For panels D and E, *Renilla* or firefly luciferase activity was measured and normalized to empty vector at 4 h postinfection or posttransfection. Data from 3 independent experiments are shown as means + SDs (two-way ANOVA, Dunnett’s multiple-comparison test; *, *P* < 0.05; **, *P* < 0.01; ***, *P* < 0.001). (F) Cellular localization of SHFL and dsRNA in mSHFL-WT or mSHFL-Zinc^mut^ Huh-7.5 cells, 24 h p.i. Cells were stained for FLAG-SHFL (green), dsRNA (red), and nuclear DNA (blue). White lines in the merged images were selected for intensity line profile analysis, shown in the right graphs.

A previous study revealed the mechanism of hSHFL restricting HIV infection by regulating programmed −1 ribosomal frameshifting (−1PRF) (23). To investigate if mSHFL retained the capacity to modulate ribosomal frameshifting, we quantified the ribosomal +1 or −1 frameshifting in the presence of ectopically expressed hSHFL and mSHFL in human liver cells (Huh-7.5) with a dual-fluorescence-based reporter assay (33). In these reporter constructs the expression of green fluorescent protein (GFP) would be constitutive; however, mCherry expression would depend on frameshifting. As a control, mCherry was also placed in the 0 reading frame. Similar to hSHFL, mSHFL also significantly reduced translation from the −1 frame; however, it did not significantly affect translation from the 0 and +1 frames (Fig. 4A). These data suggest that mSHFL retains the capacity to modulate −1 ribosomal frameshifting in human cells.

In order to determine the functional region(s) crucial for mSHFL antiviral activity, we performed a motif-searching analysis (MOTIF Search [https://www.genome.jp/tools/motif/]). We noted that all SHFL orthologues used in this study have a conserved zinc finger domain (Fig. 4B), indicating that this conserved domain may be crucial for its antiviral activity. Furthermore, our previous work revealed that the antiviral effect was strongly abrogated when the zinc finger domain on human SHFL was mutated (19). To assess the influence of this zinc finger domain on mSHFL restriction of HCV replication, we generated Huh-7.5 mSHFL Zinc^mut^ cells, which ectopically expressed mSHFL with a mutated zinc finger domain (Fig. 4B and C). We subsequently measured HCV replication using the JFH1 SGR and HCV infection using the HCV JcR2a reporter virus in the mSHFL mutant-expressing Huh-7.5 cells (Fig. 4C). In line with our previous data, mSHFL-WT restricted HCV infection and replication. In contrast, we observed complete ablation of the mSHFL antiviral activity for both HCV infection and replication in the presence of mSHFL Zinc^mut^ (Fig. 4D and E).

Zinc finger domains are typically part of protein interaction modules that mediated direct DNA or RNA binding. As HCV dsRNA is generated as a replicative intermediate in the infected cells, we explored if mSHFL colocalizes with HCV dsRNA during RNA replication. Interestingly, immunofluorescence microscopy revealed that mSHFL-WT colocalized with viral dsRNA in infected cells (Fig. 4F). In contrast, we did not observe colocalization of mSHFL Zinc^mut^ and HCV dsRNA. Collectively, these data indicate that the zinc finger domain conserved across SHFL orthologues is critical for the antiviral activity of SHFL. Moreover, the colocalization analysis suggested that mSHFL may associate with dsRNAs in a zinc finger-dependent fashion.

### Characterization of broad antiviral activity of mSHFL against RHV.

**FIG 5:**
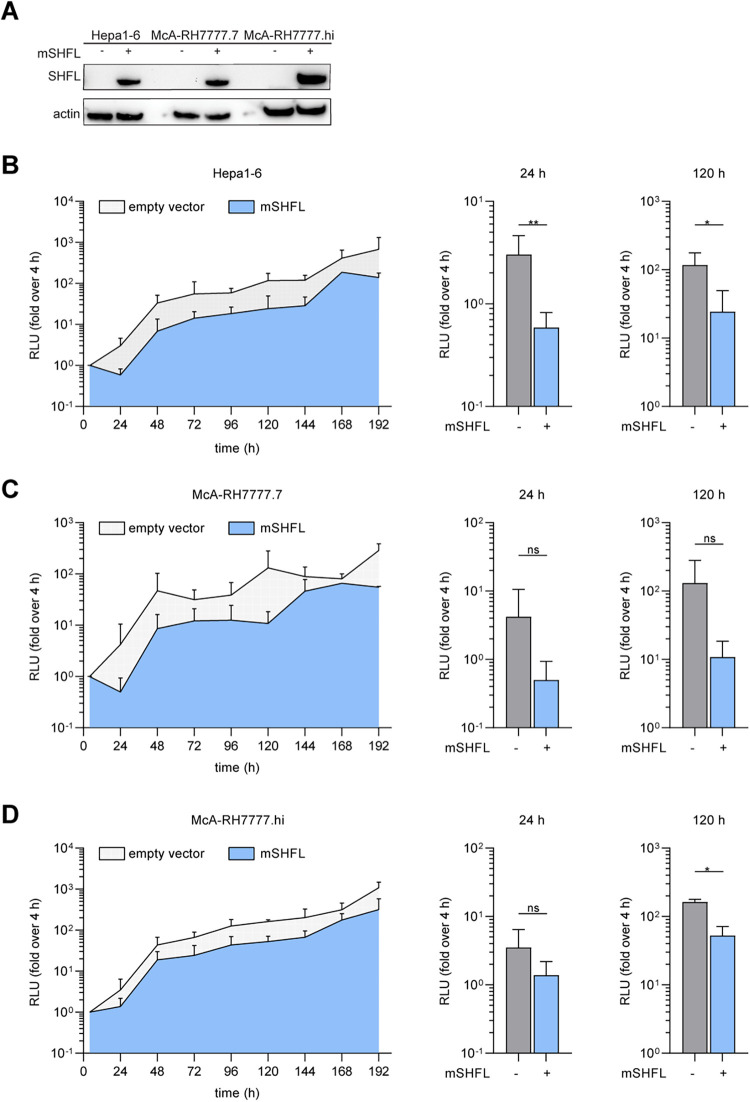
mSHFL restricts RHV replication. (A) Western blot of mouse and rat hepatoma cell lines stably expressing empty vector or mSHFL. (B to D) The left graphs show RHV replicon RNA replication in Hepa1-6 cells (B), McA-RH7777.7 cells (C), and McA-RH7777.hi cells (D) stably expressing mSHFL. Data from 3 independent experiments shown as means + SDs. The middle and right graphs show RHV replication at representative time points (24 h and 120 h posttransfection). Data from 3 independent experiments are shown as means + SDs (unpaired two-tailed *t* test, Dunnett’s multiple-comparison test; *, *P* < 0.05; **, *P* < 0.01; ***, *P* < 0.001).

Previous studies have shown that human SHFL restricts various viruses, including DENV and HCV (20, 21, 25), indicating a broad action against diverse members of the family *Flaviviridae*. Consequently, we hypothesized that mSHFL would likely restrict different members of the genus *Hepacivirus*, specifically, hepaciviruses that infect rodents. To test this hypothesis, we first generated mSHFL cell lines utilizing the mouse hepatoma cell line Hepa1-6 and two subclones from rat hepatoma cells (McA-RH7777.7 and McA-RH7777.hi) (34) (Fig. 5A). We next evaluated the replication of the rodent hepacivirus (RHV) replicon (RHV SGR) derived from the RHV-rn1 isolate (34) in mouse and rat hepatoma cell lines stably expressing mSHFL (Fig. 5B to D). The replication fitness of the RHV SGR was reduced by ectopic expression of mSHFL in Hepa1-6 and McA-RH7777.hi cells (Fig. 5B and D), providing evidence that mSHFL inhibits divergent human and rodent hepaciviruses.

## DISCUSSION

HCV infection of human liver cells elicits a strong innate immune response, which suppresses viral replication. In addition, the interaction between HCV and the innate immune response - at least in part - controls HCV cross-species transmission. Thus, a detailed understanding of these processes and identification of contributing factors which underlie the narrow species tropism of HCV will advance our understanding of HCV pathology and facilitate the stepwise development of HCV-permissive small-animal models. Ectopic expression of HCV cell entry factors CD81 (35) and OCLN (36) allows cell uptake but is insufficient to render mouse liver cells susceptible to the full HCV life cycle. This suggests that either undefined human host factors are lacking, potent murine restriction factors prevent infection, or a combination of both is responsible for the limited permissiveness of murine cells to HCV infection. Several cellular factors are known to contribute to the HCV cross-species transmission barrier to mice, including miR-122 (37), CypA (38, 39), CD302 (32), and TRIM26 (40). In this study, we characterized the potential role of mSHFL, the orthologue of the human HCV restriction factor *SHFL*, in modulating HCV infection of human and mouse hepatocytes.

Recent research revealed that SHFL restricts a diverse range of positive-stranded RNA viruses, including multiple *Flaviviridae* family members (25). Moreover, *Shfl* knockout mice exhibited increased viral replication in the central nervous system upon ZIKV infection. We show that mouse *Shfl* is endogenously expressed in mouse livers, at higher baseline levels than the majority of IRGs. *Shfl* mRNA levels are also modestly elevated upon mIFN-α or poly(I:C) treatment *ex vivo* (Fig. 1E). These findings are consistent with previous studies on the transcriptional regulation of *SHFL* in human liver cells (19, 23).

We initially investigated the evolutionary relationship between human and mouse SHFL. This assessment revealed that - compared with several classical IRGs - human and mouse SHFL orthologues are surprisingly conserved at the protein level (Fig. 1B and C). This finding suggests high levels of functional conservation in human and mouse SHFL, which may be related to their antiviral activity and/or to other physiological roles of the proteins. Given our observations that (i) mSHFL modulates ribosomal frameshifting, (ii) the conserved zinc finger domain is essential for mSHFL antiviral function, and (iii) mSHFL colocalizes with viral double-stranded RNA, we speculate that the functional properties of RNA binding and inhibition of frameshifting reflect those functional conservations. Zinc finger domains are important modules mediating protein nucleic acid interactions, and SHFL is an RNA binding protein which colocalizes with viral dsRNA (20, 21, 25, 41). Our finding that mutation of the zinc finger domain abrogates the antiviral activity of mSHFL and its colocalization with viral dsRNA (Fig. 4) are consistent with these previous reports and supports the conclusion that the zinc finger domain is important for SHFL RNA binding.

Our phylogenetic analysis incorporates a broad range of *SHFL* orthologues, including the Western clawed frog as an outgroup control, and revealed that *mShfl* and *hShfl* cluster in two distinct lineages. Interestingly, *C. porcellus Shfl* was clustered to the branch which has a closer relationship with *hShfl* (Fig. 1D). Despite this, mSHFL was more potent at restricting HCV than were all other tested orthologues (Fig. 2B and C). This suggests that the phylogenetic distance between human and nonhuman SHFL orthologues does not strictly correlate with their capacity to restrict HCV infection. Interestingly, we observed that replication of RHV is restricted by mSHFL in both rat and mouse liver cell lines (Fig. 3 and 5). This finding supports the conclusion that human and rodent SHFL orthologues restrict infection by their cognate hepaciviruses and suggests that continued exposure to these viruses may contribute to the reduced rates of SHFL evolution observed between human and mouse compared to other IRGs.

To evaluate the importance of mSHFL for prevention of cross-species transmission of HCV to mice, we modulated its expression in different murine liver cell lines. Our data show that ectopic expression of mSHFL reduced HCV RNA replication (Fig. 3A and B), whereas gene editing of *Shfl* reproducibly enhanced permissiveness to HCV infection (Fig. 3C). The effect size of manipulated mSHFL expression on HCV infection/replication in these cell culture models was modest, indicating that mSHFL contributes to the human mouse HCV species barrier but is unlikely to be the major driving force. Considering that mSHFL was robustly expressed in the livers of several mouse strains and not massively upregulated upon IFN or poly(I:C) treatment, we believe that it may play a role as an intrinsic restriction factor, functioning independently from infection-induced immune response signaling. Further clarification of the *in vivo* role of mSHFL for HCV infection of mice awaits development of tractable HCV mouse infection models. SHFL knockout animals are viable and exhibit enhanced susceptibility to ZIKV infection (25). Moreover, HCV receptor transgenic mice with ablated IFN signaling sustain low-level HCV infection. Thus, the combination of these traits may further enhance HCV susceptibility and allow for viral adaptation to ultimately create a robust HCV animal model.

## MATERIALS AND METHODS

### Cells.

Primary mouse hepatocytes (PMH) from C57BL/6 mouse were isolated as previously described (29) and cultured on a collagen-coated 6-well plate with hepatocyte culture medium (HCM; Lonza). Isolation of PMH was performed according to the guidelines of the Hannover Medical School, Germany (reference 33.19-42502-04-21/3745). Huh-7.5 (human hepatoma cells) (42), MLT-WT miR-122 (mouse liver tumor cells), MLT-MAVS^−/−^ miR-122 hhhhh (30), Hepa1-6, McA-RH7777.7, and McA-RH7777.hi (34) cells and their derived cell lines were grown in Dulbecco’s modified Eagle medium (DMEM; Gibco) supplemented with 2 mM l-glutamine (Gibco), 1% nonessential amino acids (Gibco), 100 U/mL of penicillin-streptomycin (pen-strep; Gibco), and additional 10% fetal bovine serum (FBS; Capricorn Scientific). In the case of ectopic transgene expression, medium was further supplemented with blasticidin S-hydrochloride at 5 μg/mL, puromycin at 5 μg/mL, or G418 at 750 μg/mL. All the cells were maintained at 37°C with 5% CO_2_.

Huh-7.5, MLT-WT miR-122, MLT-MAVS^−/−^ miR-122 hhhhh, Hepa1-6, McA-RH7777.7, and McA-RH7777.hi cells stably expressing SHFL orthologues were generated by lentiviral transduction. In brief, target cells were infected with lentiviral pseudoparticles carrying SHFL orthologue genes (Table 1). At 72 h after infection, 5 μg/mL of blasticidin S-hydrochloride (Fisher Scientific) was added as a selection antibiotic. The cells were ready to use in infection assays after 3 passages of culturing. Murine MLT-MAVS^−/−^ miR-122 hhhhh SHFL knockdown cells were generated by CRISPR/Cas9-mediated genome editing using lentiviral transduction (Table 1). Briefly, the target cells were infected with lentiviral pseudoparticles containing Cas9 nuclease and single guide RNA (sgRNA). Cells were cultivated for 48 h and then 100 μg/mL of hygromycin B (Invitrogen) was added as selection antibiotic. The cells were ready to use after 3 passages of culturing.

**TABLE 1:** Plasmids used in this study

Plasmid	Description
pCMV_ΔR8.74	Lentiviral expression vector including HIV-1 *gag* and *pol* with deletion of the virulence genes *env*, *vif*, *vpr*, *vpu*, and *nef* for lentivirus packaging (49)
pcZ VSV-G	Eukaryotic cytomegalovirus-based plasmid encoding vesicular stomatitis virus glycoprotein for lentivirus packaging (50)
pFK_i389Lucubineo_NS3-3′_dg_JFH	pFK vector carrying SGR encoding NS3-5B from HCV GT 2a isolate JFH1. A firefly (Photinus vulgaris) luciferase reporter gene was fused to a sequence encoding 16 amino acids from the N terminus of the HCV core protein (51).
pFKi_341_PiLuc_NS3-3′_Con1 ET	pFK vector carrying SGR encoding NS3-5B from HCV GT 1b isolate Con1. A firefly luciferase reporter gene was inserted downstream of a poliovirus internal ribosome entry site sequence (52, 53).
pLenti CRISPR v2 ccdB	Lentiviral vector encoding Streptococcus pyogenes Cas9 and a single guide RNA scaffold with the lethality gene *ccdB* flanked by BsmBI restriction enzyme sites. Kindly provided by Marc Schmidt-Supprian and Klaus Heger (54, 55).
pRHV-rn-1-FEO-lowCpG_UpA	pRHV vector carrying SGR encoding NS3-5B from rodent hepacivirus from Rattus norvegicus (RHV-rn1) and a firefly luciferase reporter gene. Kindy provided by Troels K. H. Scheel, Department of Immunology and Microbiology, University of Copenhagen, Copenhagen, Denmark (34).
pWPI_3X FLAG_BLR	Lentiviral vector plasmid encodes FLAG tag (3×FLAG tag) used for stable cell line generation under control of internal human elongation factor 1 alpha

### Plasmids.

Plasmids used in this study are listed in Table 1.

### Oligonucleotides.

Oligonucleotides used in this study are listed in Table 2.

**TABLE 2:** Oligonucleotides used in this study

Oligonucleotide	Sequence (5′–3′)	Use
Syg-*Shfl*-F	TGTAGGAAGCGTTACGAACCA	RT-qPCR
Syg-*Shfl*-R	GGAAGTTGTGGCGACACTTTG	RT-qPCR
Syg-*Isg15*-F	AGCAATGGCCTGGGACCTAAA	RT-qPCR
Syg-*Isg15*-R	AGCCGGCACACCAATCTT	RT-qPCR
Syg-*Oas1*-F	CACCCAGTGAGGGTCTCCAA	RT-qPCR
Syg-*Oas1*-R	TTGAGTGTGGTGCCTTT	RT-qPCR
Syg-*Ifnβ*-F	CTGCGTTCCTGCTGTGCTTCTCCA	RT-qPCR
Syg-*Ifnβ-R*	TTCTCCGTCATCTCCATAGGGATC	RT-qPCR
Syg-*Mx1*-F	GACCATAGGGGTCTTGACCAA	RT-qPCR
Syg-*Mx1*-R	AGACTTGCTCTTTCTGAAAAGCC	RT-qPCR
Syg-*Ifit1*-F	CTGAGATGTCACTTCACATGGAA	RT-qPCR
Syg-*Ifit1*-R	GTGCATCCCCAATGGGTTCT	RT-qPCR
Syg-*Ifit2*-F	AGTACAACGAGTAAGGAGTCACT	RT-qPCR
Syg-*Ifit2*-R	AGGCCAGTATGTTGCACATGG	RT-qPCR
Syg-*Rsad2*-F	TGCTGGCTGAGAATAGCATTAGG	RT-qPCR
Syg-*Rsad2*-R	GCTGAGTGCTGTTCCCATCT	RT-qPCR
*Shfl-g*RNA1-F	GTCGTCTCCCACCGGTCGCCACAACTTCCGGTGAGTTTCGAGACGTG	Generation of KO^*a*^ cells
*Shfl-g*RNA1-R	CACGTCTCGAAACTCACCGGAAGTTGTGGCGACCGGTGGGAGACGAC	Generation of KO cells
*Shfl*-gRNA2-F	GTCGTCTCCCACCGCGAAGATTGGCTTGCGTCAGGTTTCGAGACGTG	Generation of KO cells
*Shfl*-gRNA2-R	CACGTCTCGAAACCTGACGCAAGCCAATCTTCGCGGTGGGAGACGAC	Generation of KO cells
Scrambled-F	GTCGTCTCCCACCGCTAAGGTTAAGTCGCCCTCGGTTTCGAGACGTG	Generation of KO cells
Scrambled-R	CACGTCTCGAAACCGAGGGCGACTTAACCTTAGCGGTGGGAGACGAC	Generation of KO cells

a KO, knockout.

### Viruses.

HCV Jc1 wild-type (WT) virus and HCV JcR2a virus carrying *Renilla* reporter were cultured and generated in Huh-7.5 cells as described previously (43). The cell culture-adapted HCV (HCVcc) strain P100 was a kind gift of Esteban Domingo (CBMSO, CSIC, Madrid, Spain) and generated as described before in Huh-7.5 cells (31).

### Molecular phylogenetic analysis.

*SHFL* gene coding sequence orthologues from 15 distinct species were downloaded from Ensembl (https://www.ensembl.org/index.html) (Table 3) and used to perform phylogenetic analysis. A maximum likelihood method based on the data-specific model was implemented in MEGA (44). The bootstrap approach with 500 pseudoreplicates was applied to assess the significance of groupings. The tree with the highest log likelihood is shown (Fig. 1D), in which the significant bootstrap values (70%) are shown next to the corresponding branch. The tree was generated under a GTR+G+I model of substitution whereby the proportion of invariant sites (I) was considered during the reconstruction. The tree was drawn to scale, with branch lengths proportional to the number of substitutions per site. All positions containing gaps and missing data were eliminated. The final analysis incorporated the *SHFL* coding sequences from 15 species, and a total of 789 nucleotide positions were included in the final data set.

**TABLE 3:** SHFL orthologues used in this study

SHFL	Animo acid length	Species origin	Ensembl ID
Human SHFL	291	Homo sapiens	ENSG00000130813
Tropical clawed frog SHFL	304	*Xenopus tropicalis*	ENSXETG00000022042
Guinea pig SHFL	286	Cavia porcellus	ENSCPOG00000019761
Mouse SHFL	290	Mus musculus	ENSMUSG00000038884
Goat SHFL	291	Capra hircus	ENSCHIG00000026620
Cow SHFL	343	Bos taurus	ENSBTAG00000015636
Blue whale SHFL	287	Balaenoptera musculus	ENSBMSG00010011987
Pig SHFL	285	Sus scrofa	ENSSSCG00000013664
Horse SHFL	287	Equus caballus	ENSECAG00000024957
Dog SHFL	286	Canis lupus familiaris	ENSCAFG00845018904
Elephant SHFL	289	Loxodonta africana	ENSLAFG00000022271
Chimpanzee SHFL	291	Pan troglodytes	ENSPTRG00000010457
Rabbit SHFL	293	Oryctolagus cuniculus	ENSOCUG00000004728
Long-tailed chinchilla SHFL	288	Chinchilla lanigera	ENSCLAG00000007234
Rat SHFL	288	Rattus norvegicus	ENSRNOG00000020580

### Reverse transcription-quantitative PCR (RT-qPCR).

The total cellular RNA from PMH was extracted with a NucleoSpin RNA kit (Maherey Nagel) according to the manufacturer’s instructions. A total of 500 ng of total RNA was subsequently used for cDNA synthesis by using a PrimeScript first-strand cDNA synthesis kit (TaKaRa). The cDNA was then used to quantify the abundance of *Shfl*, *Isg15*, *Oas1*, *Ifnβ*, *Mx1*, *ifit1*, *Ifit2*, *Rsad2*, *Hprt*, and *actin* mRNA transcripts using TB Green Premix *Ex Taq* II kit (TaKaRa), respective oligonucleotides (Table 2) and the LightCycler480 system (Roche) as described previously (32). Relative mRNA expression levels of respective transcripts were calculated based on the threshold cycle (ΔΔ*C_T_*) method as described previously (45), and mouse *Hprt* was used as an internal reference gene.

### Western blot analysis.

For Western blot analysis, cells were washed 3 times with phosphate-buffered saline (PBS) and lysed with radioimmunoprecipitation assay (RIPA) buffer containing Pierce protease inhibitor minitablets (Thermo Fisher Scientific). If a reduction in viscosity was required, Benzonase nuclease (Millipore) was added to the samples, followed by incubation at 37°C for 30 min. Cell lysates were then centrifuged at high speed for 10 min, followed by transfer of the supernatant to a fresh Eppendorf tube. Quantification of the protein concentration was done using Roti-Quant (Carl Roth) or the Qubit protein assay kit (Thermo Fisher Scientific) according to manufacturer’s instructions. Equal amounts of protein were mixed with 5× sodium dodecyl sulfate-polyacrylamide gel electrophoresis (SDS-PAGE) reducing sample buffer and subsequently incubated at 95°C for 5 min. Gel electrophoresis was performed in TRIS-Glycin-SDS (TGS) or morpholineethanesulfonic acid (MES) running buffer. Separated proteins were transferred to polyvinylidene difluoride (PVDF) membranes (0.45 μm) by semidry electroblotting technique. After blotting, PBS containing 0.1% Tween 20 (PBS-T) and 5% low-fat milk powder was used to block unspecific proteins on the PVDF membrane by 2 h of incubation at room temperature under mild shaking conditions. The membrane was then incubated with one of the primary antibodies anti-FLAG M2 (F3165 or F1804, 2 μg/mL; Sigma-Aldrich), anti-β-actin AC-74 (A2228, 1 μg/mL; Sigma-Aldrich), anti-ISG15 (number 2743, 1:500; Cell Signaling), and anti-C19orf66 (Ab122765, 0.8 μg/mL; Abcam) diluted in blocking buffer at 4°C overnight. The next day, the membrane was washed 3 times in PBS-T before a horseradish peroxidase (HRP)-conjugated secondary antibody diluted in blocking buffer was added to the membrane and incubated for 1 h at room temperature. The additionally used HRP-conjugated anti-β-actin AC-15 (A3854, 1:5,000; Sigma-Aldrich) was incubated similarly to secondary antibodies for 1 h at room temperature. Membranes were again washed 3 times in PBS-T to remove unbound secondary antibodies. Subsequently, proteins were detected using an ECL Prime Western blotting detection reagent (GE Healthcare) or SuperSignal West Femto maximum-sensitivity substrate (Thermo Scientific) and a ChemoCam Western blot imaging system (Intas) or the Fusion FX7 EDGE imager (Vilber).

### *In vitro* RNA transcription and electroporation.

pFK_i389Lucubineo_NS3-3′_dg_JFH, pFKi_341_PiLuc_NS3-3′_Con1 ET, and pRHV-rn-1-FEO-lowCpG_UpA plasmids (Table 1) from validated clones were used for *in vitro* transcripts and electroporation-based transfection as described previously (28). The cells were subsequently seeded into 96-well plates for further analysis.

### HCV infection assays.

HCV Jc1 WT, HCVcc P100, and HCV reporter virus JcR2a were used for infectivity kinetic evaluation. At 4 h p.i., the DMEM was removed and fresh medium was added after 3 PBS washes. For HCV Jc1 WT and HCVcc P100, infectivity was calculated via limiting-dilution assays to determine 50% tissue culture infective doses (TCID_50_) per milliliter (46). For JcR2a, cells were lysed with H_2_O prior to *Renilla* luciferase measurement.

HCV Jc1 WT virus was used for immunofluorescence staining and for infection of the Huh-7.5 mSHFL overexpression cell line or Huh-7.5 mSHFL zinc finger mutant cell line at a multiplicity of infection (MOI) of 1. At 24 h p.i., the cells were fixed before further analysis.

MLT-MAVS^−/−^ miR-122 hhhhh stably expressing SHFL or *Shfl* gene-edited cells were infected with HCV P100 at an MOI of 2. At 4 h p.i., the cells were washed 3 times with PBS and fresh medium was added. Cell culture supernatant was collected at the indicated time points postinfection. The supernatant was then used to titer the infectious virus release on Huh-7.5 cells using the TCID_50_ assays as described previously (46).

### Firefly and *Renilla* luciferase assays.

A total of 35 μL of luciferase lysis buffer (0.1% Triton X-100, 25 mmol/L of glycylglycine, 15 mmol/L of MgSO_4_, 4 mmol/L of EGTA tetrasodium, and 1 mmol/L of dithiothreitol [pH 7.8]) (firefly luciferase assay) or H_2_O (*Renilla* luciferase assay) was added to a 96-well plate. To quantify the replication activity of firefly reporter HCV SGR or RHV SGR, 20 μL of cell lysate was mixed with 72 μL of assay buffer. A total of 40 μL of fresh luciferin solution (200 μmol/L of d-luciferin substrate, 25 mmol/L of glycylglycine [pH 8]) was loaded to samples using the microplate reader LB 960 plate luminometer. To detect infection activity of *Renilla* reporter HCV JCR2a, 60 μL of freshly prepared coelenterazine substrate (1 μM in PBS; PJK GmbH) was loaded to 20 μL of cell lysate pre-filled in a 96-well plate by the microplate reader LB 960 plate luminometer. Bioluminescence as a result of oxidation of d-luciferin substrate or coelenterazine by firefly or *Renilla* luciferase, respectively, was then quantified by the devices.

### Quantification of HCV ribosomal frameshifting.

Huh-7.5 cells stably expressing hSHFL or mSHFL, as well as control cells, were transiently transfected with either the in-frame control or a frameshifting construct as described previously (33, 47, 48). Transfection with this reporter leads to expression of enhanced GFP (EGFP) when the 0 reading frame is translated. Translation of mCherry is dependent on frameshifting. Thus, the ratio between mCherry and EGFP directly correlates to frameshifting efficiency. As a normalization control, we used an in-frame construct lacking a frameshift site, leading to the expression of EGFP and mCherry in equal ratios. Using flow cytometry (Novocyte Quanteon), EGFP^+^ cells were analyzed for the ratio between mCherry and EGFP. Frameshifting efficiency was calculated as the mCherry/EGFP ratio in the frameshifting reporter construct relative to the mCherry/EGFP ratio for the control construct.

### Immunofluorescence staining and confocal microscopy assay.

To stain mSHFL upon HCV infection, Huh-7.5 cells stably expressing mSHFL were infected with 748 TCID_50_/well of HCV JcR2a virus. At 4 h p.i., cells were washed 3 times with PBS before fresh medium was added. At 48 h p.i., cells were fixed for further microscopy analyses. To detect the colocalization between HCV viral dsRNA and mSHFL, Huh-7.5 cells stably expressing mSHFL or a zinc finger mutant were infected with Jc1 WT virus at an MOI of 1. At 4 h p.i., cells were washed 3 times with PBS and fresh medium was added. At 24 h p.i., the cells were fixed for immunofluorescence analysis. For this, 300 μL/well of 3% paraformaldehyde (PFA) was added to 24-well plates (coverslips seeded) and incubated at room temperature for 10 min. Cells were then washed 3 times with PBS and preserved at 4°C until immunofluorescence staining was performed. Fixed cells were permeabilized with 0.5% Triton X-100 for 5 min. After three washing steps with PBS and blocking with 5% goat serum for 1 h at room temperature, primary antibodies anti-C19orf66/SHFL (1.5 μg/mL; Abcam), anti-HCV E2 (CBH23; 3 μg/mL), anti-dsRNA (2 μg/mL; Jena Bioscience) diluted in PBS containing 5% goat serum (Sigma-Aldrich) were incubated at room temperature for 1 h, followed by 3 washing cycles with PBS. Cells were incubated with 2 μg/mL of secondary antibodies (Alexa Fluor 488-coupled goat anti-rabbit IgG, Alexa Fluor 568-coupled goat anti-human IgG and Alexa Fluor 647-coupled donkey anti-mouse IgG; Invitrogen) at room temperature for 1 h, followed by three additional washing cycles with PBS to remove unbound antibodies. The nuclei were stained at room temperature for 5 min with 4′,6-diamidino-2-phenylindole (DAPI; Thermo Fisher), followed by 3 additional washing cycles with PBS. Afterwards, glass coverslips were mounted on glass slides with Prolong gold antifade (Thermo Fisher) and stained cells were analyzed using a confocal microscope (FV1000; Olympus) at a magnification of ×20 or ×100.

### Cell viability assay.

A total of 2.5 × 10^4^ cells were seeded in a 48-well format. At the indicated time points, cells were trypsinized in 100 μL of trypsin-EDTA and then 400 μL of DMEM complete medium was added to each well before cells were counted in a hemocytometer at a 1:1 ratio with trypan blue.

### Statistical analysis.

Statistical significance of differences of means was calculated using either unpaired two-tailed *t* test or one-way or two-way analysis of variance (ANOVA) followed by Dunnett’s multiple-comparison test. *P* values of <0.05 were considered significant. Data presented on log scales were transformed [*y* = log(*y*)] prior to statistical analysis. Utilized tests are specified in the corresponding figure legends.
